# Targeting the receptor tyrosine kinase RET in combination with aromatase inhibitors in ER positive breast cancer xenografts

**DOI:** 10.18632/oncotarget.11826

**Published:** 2016-09-02

**Authors:** Elena Andreucci, Paola Francica, Antony Fearns, Lesley-Ann Martin, Paola Chiarugi, Clare M. Isacke, Andrea Morandi

**Affiliations:** ^1^ Department of Experimental and Clinical Biomedical Sciences, University of Florence, Florence, Italy; ^2^ The Breast Cancer Now Toby Robins Research Centre, The Institute of Cancer Research, London, United Kingdom; ^3^ Tuscany Tumor Institute (ITT) and Excellence Centre for Research, Transfer and High Education DenoTHE, Florence, Italy; ^4^ Current address: Department of Clinical Research, Radiation Oncology Laboratory, University of Bern, Bern, Switzerland; ^5^ Current address: The Francis Crick Institute, Mill Hill Laboratory, The Ridgeway, London, United Kingdom

**Keywords:** RET, GDNF, endocrine therapy, aromatase inhibitors, resistance

## Abstract

The majority of breast cancers are estrogen receptor positive (ER+). Blockade of estrogen biosynthesis by aromatase inhibitors (AIs) is the first-line endocrine therapy for post-menopausal women with ER+ breast cancers. However, AI resistance remains a major challenge. We have demonstrated previously that increased GDNF/RET signaling in ER+ breast cancers promotes AI resistance. Here we investigated the efficacy of different small molecule RET kinase inhibitors, sunitinib, cabozantinib, NVP-BBT594 and NVP-AST487, and the potential of combining a RET inhibitor with the AI letrozole in ER+ breast cancers. The most effective inhibitor identified, NVP-AST487, suppressed GDNF-stimulated RET downstream signaling and 3D tumor spheroid growth. Ovariectomized mice were inoculated with ER+ aromatase-overexpressing MCF7-AROM1 cells and treated with letrozole, NVP-AST487 or the two drugs in combination. Surprisingly, the three treatment regimens showed similar efficacy in impairing MCF7-AROM1 tumor growth *in vivo*. However *in vitro*, NVP-AST487 was superior to letrozole in inhibiting the GDNF-induced motility and tumor spheroid growth of MCF7-AROM1 cells and required in combination with letrozole to inhibit GDNF-induced motility in BT474-AROM3 aromatase expressing cells. These data indicate that inhibiting RET is as effective as the current therapeutic regimen of AI therapy but that a combination treatment may delay cancer cell dissemination and metastasis.

## INTRODUCTION

Endocrine therapies have shown to be effective in patients that express estrogen receptor-α (ERα, called hereafter ER). However, a significant portion of patients display *de novo* resistance or will develop resistance after an initial response. In postmenopausal patients, aromatase inhibitors (AIs), which block the conversion of androgens to estrogens, are the first-line treatment choice [1]. We, along with others, have demonstrated previously that a major cause of AI resistance is growth factor receptor activation that, via the PI3K/AKT/mTOR or MAPK pathways, drives ligand-independent ER activation [2–6]. These findings have been exploited clinically by combining AIs with mTOR [7, 8] or PI3K/AKT (NCT01437566) [9, 10] inhibitiors.

We have reported that activation of the REarranged during Transfection (RET) receptor tyrosine kinase by its ligand GDNF decreases response of ER+ breast cancer cells to endocrine therapy, including AIs, and that the transcriptional signature of RET downstream signaling has both prognostic and predictive value in breast cancer [4, 11–13]. Accordingly, the combination of the AI letrozole with the RET inhibitor NVP-BBT594 is more effective at suppressing GDNF-induced proliferation of RET+ ER+ breast cancer cells than either monotherapy [12, 14].

In the current study, we first examined *in vitro* a number of small molecule inhibitors known to target RET that could be used in combination with an AI *in vivo*. We then chose the most effective inhibitor to assess its efficacy when combined with letrozole in an *in vivo* AI-sensitive breast cancer xenograft model.

## RESULTS

### Impact of different small kinase inhibitors on GDNF-induced RET signaling in ER+/RET+ MCF7 cells

**Figure 1:**
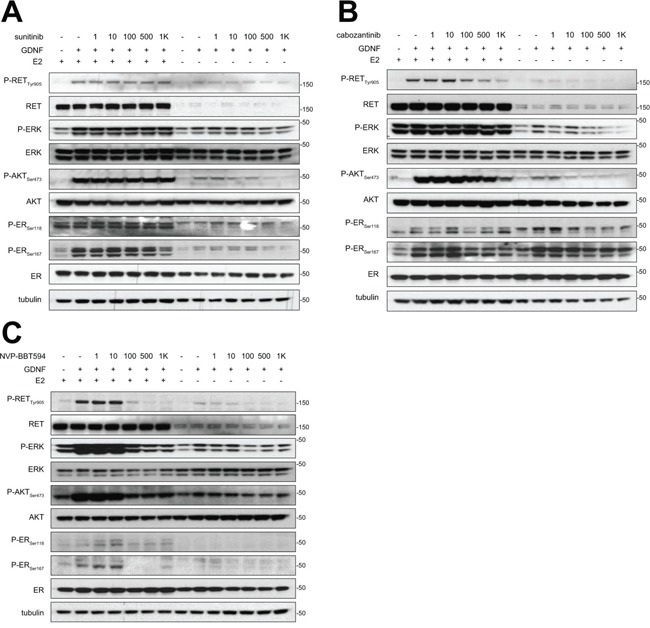
NVP-BBT594 impairs RET downstream signaling at nanomolar concentrations in a dose dependent manner Wild-type MCF7 cells were grown in complete medium (E2+) or in E2-deprived DCC medium (E2-) for 3 days, serum-starved for the last 24 hours and pre-treated with the indicated concentrations of **A.** sunitinib, **B.** cabozantinib, or **C.** NVP-BBT594 for 90 minutes before 30 minutes GDNF (20 ng/ml) stimulation. Total cell lysates were subjected to western blotting using the indicated antibodies. Tubulin was used as a loading control. Molecular size markers are in kDa.

**Figure 2:**
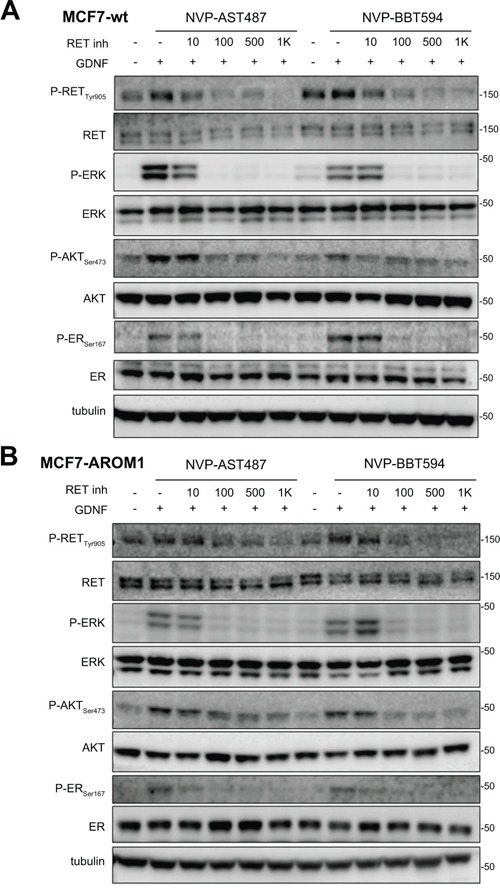
NVP-BBT594 and NVP-AST487 have comparable inhibitory effects on RET downstream signaling in both wild-type and aromatase-expressing (AROM1) MCF7 cells **A.** Wild-type MCF7 cells were serum-starved for 24 hours before pre-treating with NVP-AST487 or NVP-BBT594 for 90 minutes before GDNF (20 ng/ml) stimulation for 30 minutes. For the experiments performed in the absence of E2, wild-type MCF7 cells were 3 day E2 deprived in DCC medium before serum starvation. **B.** MCF7-AROM1 cells were E2 deprived for 3 days in DCC medium and stimulated with androstenedione (10 mM) for the last 24 hours. The cells were then serum-starved for a further 24 hours and pre-treated with the indicated concentrations of NVP-AST487 or NVP-BBT594 for 90 minutes before GDNF (20 ng/ml) stimulation for 30 minutes. Total cell lysates were subjected to western blotting using the indicated antibodies. Tubulin was used as a loading control. Molecular size markers are in kDa.

We have demonstrated that GDNF-dependent RET signaling promotes phosphorylation of ER and that, in these cells, ER transcriptional activity is blocked by siRNA-mediated downregulation of RET expression [4]. Further, the inhibitor NVP-BBT594 has been shown to impair RET signaling within nanomolar concentrations *in vitro*, however this compound showed toxicity *in vivo* [12]. Consequently, we first compared the efficacy of NVP-BBT594 with other small molecule RET inhibitors [11]. Three day E2-deprived wild-type MCF7 cells were treated with the kinase inhibitors sunitinib (Figure 1A), cabozantinib (XL-184) (Figure 1B) and NVP-BBT594 (Figure 1C) at increasing concentrations and stimulated with 20 ng/ml GDNF in presence or absence of E2. Since RET has been shown to be an ER-dependent gene [14], the presence of E2 in the culture medium enhanced RET expression resulting in a stronger activation of GDNF-induced RET downstream signaling (Figure 1). Of the compounds used, NVP-BBT594 showed the highest suppression of GDNF-induced RET signaling, as assessed by RET, ERK1/2, AKT and ER phosphorylation. However, as stated above, NVP-BBT594 was unsuitable for extending these studies into *in vivo* models due to its *in vivo* toxicity. Consequently, we extended our studies to another RET inhibitor NVP-AST487, known to be well tolerated by mice [15, 16]. Western blot analysis revealed that NVP-AST487 and NVP-BBT594 have comparable RET inhibitory activity in wild-type MCF7 cells (Figure 2A). Importantly, similar results were obtained in MCF7 derivatives with stable expression of aromatase, MCF7-AROM1 cells (Figure 2B), which provides a model of an AI sensitive breast cancer cells. In these experiments, MCF7-AROM1 cells were deprived of E2 for 3 days and then treated with androstenedione, which is converted into estrogen by the aromatase enzyme.

### NVP-AST487 reduces GDNF-induced MCF7-AROM1 cell growth in 3D *in vitro* assays

**Figure 3:**
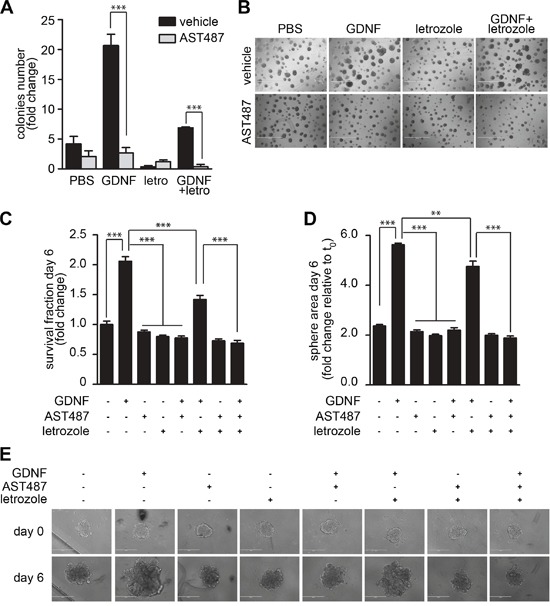
NVP-AST487 reduces GDNF-induced MCF7-AROM1 cell growth in 3D *in vitro* assays MCF7-AROM1 cells deprived of E2 for 3 days in DCC medium were stimulated with 10 nM androstenedione and then maintained with or without GDNF (20 ng/ml) in the presence or absence of NVP-AST487 (100 nM) and/or letrozole (100 nM) as indicated. Cells were subjected to **A, B.** colony formation assays on Matrigel (10 days), **C.** cell viability assay (6 days) or **D.** spheroid formation assays performed in non-adherent conditions (6 days). Data represent mean +SEM, n=3 independent experiments. (A) Two-way ANOVA, Bonferroni corrected ***, *P*<0.001. (B) Representative pictures of the assay described in A. Scale bar, 1 mm. C and D, One-way ANOVA; Bonferroni corrected; **, *P*<0.01; ***, *P*<0.001. **E.** Representative phase contrast images of spheres quantified in D. Scale bar: 400 μM.

Next, we evaluated whether the inhibitory effects exerted by NVP-AST487 on MCF7-AROM1 cells alter tumor cell growth in two independent physiologically relevant *in vitro* assays. In the first, MCF7-AROM1 cells were subjected to a colony formation assay on a layer of thick Matrigel (Figure 3A, 3B) and in the second, cells were grown as three dimensional (3D) tumor spheroids in non-adherent conditions (Figure 3C-3E). In both settings, MCF7-AROM1 cells were stimulated with androstenedione in the presence or absence of GDNF, NVP-AST487 and/or letrozole. When plated onto thick Matrigel, GDNF-stimulated MCF7-AROM1 cells formed a significantly larger number of colonies than vehicle treated cells. Importantly, NVP-AST487 treatment was more effective than letrozole at blocking the GDNF-mediated increase in colony formation. Comparable results were obtained in the tumor spheroid formation assay (Figure 3C-3E). Indeed, both the cell viability (Figure 3C) and the size of tumor spheres (Figure 3D) were significantly increased by GDNF treatment and this GDNF-mediated increase was fully blocked by NVP-AST487 treatment. In contrast, treatment with letrozole was only partially effective in impairing these GDNF-induced effects, suggesting that in a GDNF-enriched microenvironment (i.e. an environment enriched in GDNF secreting stromal cells [17, 18]) endocrine therapy alone may not be sufficient to impair RET-dependent tumor growth. We therefore evaluated if a combinatory therapeutic approach was of relevance in an *in vivo* setting.

### Combinatory treatment of NVP-AST487 and letrozole is not superior to monotherapies in reducing primary tumor growth

**Figure 4:**
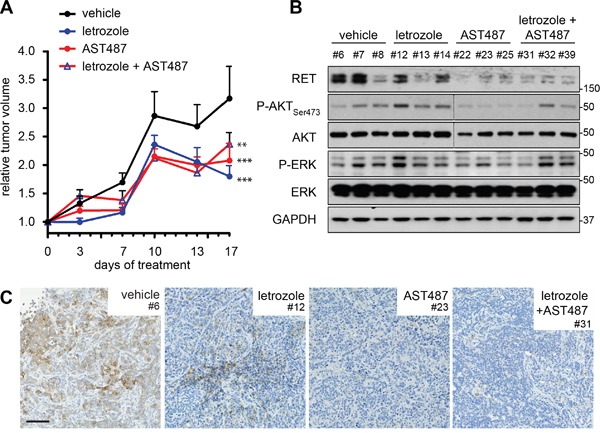
Combination treatment of NVP-AST487 and letrozole is not superior to monotherapies in reducing primary tumor growth Ovariectomized mice under androstenedione support were inoculated with MCF7-AROM1 cells. Following randomization, mice were treated daily with vehicle, letrozole, NVP-AST487 or letrozole plus NVP-AST487 as described in the Methods (n = 10 mice per group). **A.** Tumor volumes show as fold change relative to day 1 of treatment +SEM (2-way ANOVA, Tukey's corrected; **, *P*<0.01, ***, *P*<0.001). At the end of the experiment, tumors were halved for **B.** extraction of total lysates (n = 3 tumors per treatment group) for western blotting as indicated. GAPDH was used as a loading control. Molecular size markers are in kDa. Dotted line indicate two separate gels, run in parallel and exposed for equal lengths of time and **C.** RET immunohistochemistry. Representative images are shown. Scale bar, 200 μm.

MCF7-AROM1 cells were injected subcutaneously into immunocompromised mice where they formed tumors under androstenedione support, due to aromatase-mediated conversion into estrogen. Tumor-bearing mice were treated with AI letrozole, NVP-AST487 or the combination and sacrificed after three weeks (Figure 4A). NVP-AST487, either when administered alone or in combination with letrozole, clearly impaired the RET signaling pathway as monitored by the reduction in RET protein levels and a decrease in AKT phosphorylation (Figure 4B). Importantly, ERK phosphorylation was moderately suppressed by NVP-AST487, highlighting a discrepancy between the *in vitro* and the *in vivo* experimental settings, potentially due to other RET-independent ERK activating signaling pathways activated by the tumor microenvironment inputs. The decrease in RET protein levels induced by NVP-AST487 treatment was also confirmed at cellular level by immunohistochemistry, indicating that NVP-AST487 was able to access the RET+ cell compartment of the tumor (Figure 4C and Supplementary Figures S1 and S2). Moreover, NVP-AST487 treatment alone impaired tumor growth. In these experiments it is important to note that there was no exogenous GDNF treatment indicating that in an *in vivo* setting RET signaling is active in these ER+ breast cancer xenografts. In contrast to results obtained in 3D *in vitro* assays, RET inhibition and letrozole treatment as monotherapies showed comparable effects in impairing xenograft growth and, moreover, the combination of NVP-AST487 and letrozole had no additional effect on tumor growth (Figure 4A).

These results are consistent with those obtained by Gattelli *et al*., using a RET+ syngeneic xenograft model of J110 mouse mammary tumor cells in FVB mice. J110 tumor-bearing mice were treated with the NVP-AST487 inhibitor in the presence or absence of fulvestrant, an endocrine therapy that acts by targeting ER for degradation [16]. Again, the monotherapies had comparable effects in reducing primary tumor growth, but there was no enhanced effect when the two compounds were used in combination. Taken together these two studies, using distinct *in vivo* breast cancer models, indicate that RET signaling in tumor cells *in vivo* promotes tumor growth. Furthermore, inhibiting either RET signaling or ER signaling (either by blocking E2 production or promoting ER degradation) is comparable in reducing primary tumor growth. However, in the Gattelli study, RET inhibition combined with fulvestrant treatment led to a significant reduction in spontaneous metastasis, compared to either treatment alone [16].

### NVP-AST487, but not letrozole, blocks GDNF-induced MCF7-AROM1 cell migration

**Figure 5:**
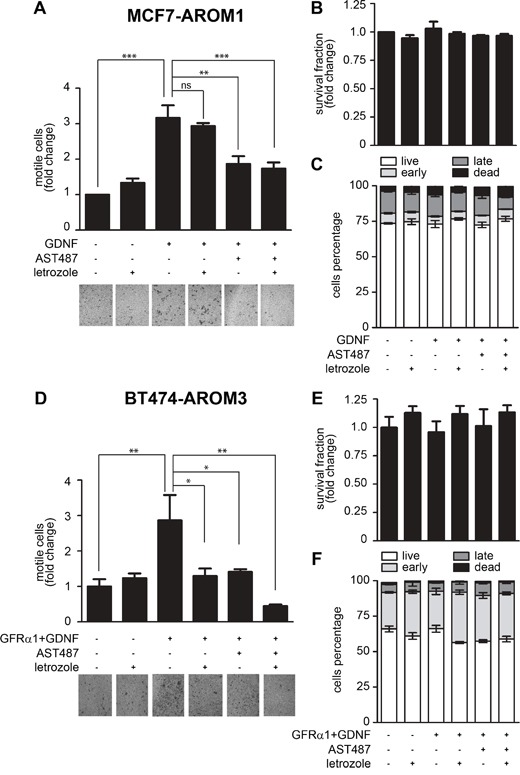
NVP-AST487, but not letrozole, reduces GDNF-induced MCF7-AROM1 cell motility MCF7-AROM1 **(A-C)** and BT474-AROM3 **(D-F)** cells deprived of E2 for 3 days in DCC medium were stimulated with androstenedione (10 nM) in the presence or absence of letrozole (100 nM), GFRα1 (100 ng/ml), GDNF (20 ng/ml) and/or NVP-AST487 (100 nM). (A, D) Cell motility in Transwells was measured after 48 hours. One-way ANOVA, Bonferroni's corrected, **, *P*<0.01, ***, *P*<0.001. Representative images of DiffQuick strained Transwell filters are shown. 4X magnification. (B, E) Cell viability was measured after 48 hours. Data represent mean +SEM, n=3 independent experiments. One-way ANOVA, Bonferroni's corrected, ns: not significant. (C, F) Apoptosis was measured using the PI/AnnexinV assay. Bar graphs show mean percentage +SEM of cells that are alive, undergoing early or late apoptosis and cells that are dead. One-way ANOVA revealed no significant differences between the groups.

Since MCF7-AROM1 cells do not spontaneously metastasize in mice, we could not directly assess the ability of NVP-AST487 to impair metastatic dissemination. Therefore, we investigated the effect of NVP-AST487 on MCF7-AROM1 cells motility *in vitro*, using the Boyden chambers assay. Treatment with GDNF resulted in a significant increase in MCF7-AROM1 cell motility. Inhibition of aromatase activity with letrozole had no inhibitory effect on the migration of GDNF-treated cells, but this enhanced cell motility was fully reverted by NVP-AST487 treatment (Figure 5A). Importantly, in the time-frame of the cell migration experiments, neither monotherapies nor combination treatment impaired cell viability in 2D culture (Figure 5B) or increased cell apoptosis (Figure 5C), indicating that the reduction in cell motility observed in the presence of NVP-AST487 was due to direct inhibition of RET. These data were confirmed in an additional cell line, the ER+/HER2+ aromatase-expressing BT474-AROM3 cells. These RET-positive cells express 1-log lower levels of the RET co-receptor GFRα1 when compared to MCF7 cells (not shown) and, importantly, GFRα1 levels are influenced by letrozole addition (Supplementary Figure S3). Consequently, soluble GFRα1 was added for optimal GDNF-induced RET signaling activation (Supplementary Figure S3) as previously described [12]. In Transwell migration assays, GDNF-induced RET activation resulted in a significant increase in BT474-AROM3 cell motility (Figure 5D), which was blocked to a similar extent by both letrozole and NVP-AST487 treatment. Importantly, combining the two compounds resulted in a greater inhibition of GDNF-induced BT474-AROM3 cell motility (One-way ANOVA, Bonferroni's corrected, *P*<0.05; Figure 5D), without significantly impairing cell viability (Figure 5E) or promoting apoptosis (Figure 5F).

## DISCUSSION

In this study, we demonstrate that NVP-AST487 and NVP-BBT594 have comparable potencies as inhibitors of GDNF-induced RET signaling in ER+ breast cancer cells *in vitro*. NVP-AST487 was chosen for *in vivo* validation as it has a superior safety profile in mice. Notably, we show that suppressing GDNF/RET signaling *in vivo* with NVP-AST487 is comparable in efficacy to AI treatment, the standard of care for postmenopausal ER+ breast cancer that is modeled in this experimental setting.

Breast cancer cells can escape the inhibitory effects of endocrine therapies by increasing ER activity independently of estrogen [5]. We, along with others, have reported that the receptor tyrosine kinase RET plays an important role in endocrine therapies resistance in ER+ breast cancer cells by promoting ER phosphorylation, cell proliferation, and cell survival [4, 11, 12, 16]. Further, it has been recently demonstrated that RET expression and activation may also be physiologically relevant in triple negative (negative for ER, progesterone and HER2 receptor expression) [19] and HER2 positive breast cancers [19, 20]. Interestingly, derivatives of the cytotoxic agent maytansine, a microtubule-interfering drug, conjugated to an anti-RET antibody show dose-dependent antitumor activity in RET expressing breast cancer xenograft models [20]. However, RET is expressed in the adult peripheral nervous system and treatment with this agent is also associated with on-target neuropathy. As a consequence, there remains a need for selective RET inhibitors that could be of potential clinical application in breast cancer but with reduced toxicity. Encouraging data has come from treating *in vivo* breast cancer patient-derived xenografts (PDXs) with the small kinase inhibitor vandetanib [19]. Although vandetanib reduced RET activation and promoted tumor regression in a subset of PDXs, it is important to note that this inhibitor also targets other tyrosine kinases, in particular VEGFR and EGFR, and that vandetanib induced an anti-angiogenic response in all PDX tumors independent of their RET or EGFR expression levels.

Since RET expression and activation is associated with endocrine therapy resistance, there is a rationale for combining endocrine agents with RET inhibitors. Spanheimer and coworkers have recently reported that the selective ER antagonist tamoxifen and vandetanib had comparable efficacy in reducing MCF7 tumor growth in *in vivo*. Furthermore, combining the two compounds was significantly more effective in reducing tumor growth than either agent alone [21]. However, this result is discordant with that of Gattelli and coworkers who investigated the effects of combining a RET inhibitor with endocrine therapy *in vivo* using the J110 breast cancer murine model [16]. They reported in J110 xenografts, no additive effect of combining a RET inhibitor with either fulvestrant or tamoxifen [16], consistent with the findings reported here using an AI-sensitive human xenograft model. Importantly, in the J110 model the authors reported a strong effect of the RET inhibition on reducing metastatic dissemination, particularly when combined with fulvestrant or tamoxifen, indicating that RET activation can both promote tumor growth and represent an important requirement for J110 tumor cell dissemination.

In the current study, we demonstrate that, as previously reported [22, 23], MCF7-AROM1 xenografts are sensitive to the AI letrozole. In this experiment, it is notable that the concentration of letrozole used (i.e. 1 mg/kg/day) slowed tumor growth. However, the same concentration in alternative letrozole-sensitive ER+ xenograft model was sufficient to induce complete tumor remission [24]. These differences may be due to the different cell models used, different levels of aromatase expression and/or to a different androgen-dependency that these models may display.

Since MCF7-AROM1 cells do not form metastases in mice, we could not directly assess the ability of NVP-AST487 to impair metastatic dissemination in when used in combination with an AI. However, it was striking that in *in vitro* cell migration assays, treatment with letrozole alone had no inhibitory effect on GDNF-induced MCF7-AROM1 cell motility whereas NVP-AST487 treatment resulted in an effective impairment. Interestingly, in an alternative model, letrozole treatment reduced BT474-AROM3 cell motility but full blockade was achieved only by combining the RET inhibitor and letrozole.

In summary, the data presented support the hypothesis that RET inhibitors, such as NVP-AST487, could both impair primary tumor growth and tumor dissemination, thereby providing a potential therapeutic advantage when used in combination with AIs in post-menopausal ER+ breast cancers.

## MATERIALS AND METHODS

### Cell lines and assay

All cell lines were STR (PCR/short tandem repeat) profiled by DNA Diagnostic Centre (DCC, London, UK). Wild-type MCF7 human breast cancer cells were maintained in phenol red-free RPMI 1640 supplemented with 10% fetal bovine serum (FBS, Euroclone), 2 mM glutamine, and 1 nM 17-β estradiol (E2, Sigma). MCF7 cells expressing full-length human aromatase MCF7-AROM1 [22] were employed as a model of AI-sensitive cells, and maintained in DMEM supplemented with 10% FBS, 2 mM L-glutamine, and 1 mg/mL Geniticin/G418 (Invitrogen). For estrogen deprivation, wild-type or AROM1 MCF7 and BT474-AROM3 cells were cultured for 3 days in phenol red-free RPMI-1640 medium plus 10% dextran charcoal-treated (DCC) FBS and 2 mM glutamine (DCC medium). After 72 h, MCF7-AROM1 and BT474-AROM3 cells were treated with 10 nM of androstenedione (Sigma), which is converted into estrogens by aromatase, and after 24 h with the AI letrozole (Sigma). Cell viability, Western blot analysis, 3D colony forming and Annexin V/PI apoptosis assays were as previously reported [4, 12, 25].

### Compounds

NVP-AST487 (N-[4-[(4-ethyl-1-piperazinyl)methyl]-3-(trifluoromethyl)phenyl]-N'-[4-[[6-(methylamino)-4-pyrimidinyl]oxy]phenyl]-urea) and NVP-BBT594 (5-[[6-(acetylamino)-4-pyrimidinyl]oxy]-2,3-dihydro-N-[4-[(4- methyl-1-piperazinyl) methyl]-3-(trifluoromethyl)phenyl]-1H-indole-1-carboxamide) were provided by Novartis Pharmaceuticals, Basel (Switzerland). Cabozantinib and sunitinib were purchased from Tocris Bioscience.

### Tumor spheroids growth assay

1 × 10^3^ MCF7-AROM1 cells per well were seeded in 96-well ultra-low attachment plates in phenol red-free RPMI supplemented with 10% DCC FBS and 10 nM androstenedione [26]. After 24 hours, assembled spheroids were stimulated with 20 ng/ml GDNF (BD Biosciences) and treated with 100 nM NVP-AST487 and/or 100 nM letrozole. Medium was replaced every 3 days. Spheroid growth was monitored by collecting images of the spheroids and subsequent size quantification using ImageJ software. Cell viability was measured at day 6 with CellTiter-Glo luminescent assay according to the manufacturer's instructions (Promega, Madison, WI, USA) using a Victor 2V Multilabel HTS counter (Wallac-Perkin Elmer, Herts, UK).

### Colony proliferation assay

Growth factor reduced and phenol red-free Matrigel (BD Biosciences, Oxford, UK) was diluted 1:1 in PBS and 0.2 mL per well was plated into 24-well tissue culture dishes and left at 37°C for 2–3 h. Next, 1 × 10^4^ cells were seeded per well and cultured overnight. The following day, cells were stimulated with GDNF (20 ng/mL) and treated with letrozole, NVP-AST487 or the combination as indicated. Medium was replaced every 3 days and colonies larger than 200 μm were counted at day 10.

### *In vitro* transwell motility assay

For motility assay, cells were serum starved overnight and seeded into the upper chamber of 8 μm-pore Transwells (Corning) in serum-free growth medium, with or without the indicated compounds. Cells were allowed to migrate towards complete growth medium for 48 hours. Non-moving cells were removed mechanically and the underside of the Transwells was stained with DiffQuick solution (BD Biosciences) as previously reported [27]. Chemotaxis was evaluated by counting the cells migrated to the lower surface of the filters (six randomly chosen fields).

### *In vivo* experiments

MCF7-AROM1 xenografts were established as previously described [28]. In brief, 1 × 10^7^ MCF7-AROM1 cells were inoculated subcutaneously into ovariectomized female Ncr Foxhead nude 6- to 8-week-old mice (Harlan). Throughout the course of the experiment, mice were treated daily with androstenedione via intradermal injection (100 μg/day). Tumors were grown to ~8 mm diameter and then the mice were assigned to the following treatment groups. All treatments were given daily. Vehicle, 10% N-methyl-pyrollidone (NMP)/90% polyethylene glycol (PEG300); letrozole, 1 mg/kg in 150 μL of 10% NMP/90% PEG300; NVP-AST487, 50 mg/kg/d; combination, combination of letrozole and NVP-AST487. Tumor growth was assessed by caliper measurements of the two largest tumor diameters. Volumes were calculated according to the formula: a×b^2^×π/6, where a and b are orthogonal tumor diameters. All animal work was carried out with UK Home Office approval.

### Immunostaining

For RET immunohistochemistry (IHC), antigen retrieval was carried out by microwave irradiation for 5 minutes at full power (900 W) in citrate buffer pH 6.0. Anti-Ret (E1N8X) XP antibody (Cell Signaling technology) was optimized to give robust IHC staining of formalin-fixed paraffin-embedded material and was applied at 1/100 dilution (see Supplementary Figure S1). The stained slides were then scanned on a whole slide scanner (Nanozoomer 2.0-HT, Hamamatsu, Japan).

### Statistical analysis

Statistical analysis was conducted using GraphPad Prism Software as reported in the figure legends and Results.

## SUPPLEMENTARY MATERIALS FIGURES


