# Supplementary material for: Targeting the receptor tyrosine kinase RET in combination with aromatase inhibitors in ER positive breast cancer xenografts
Source: Oncotarget. 2016 Sep 2;7(49):80543–53. doi: 10.18632/oncotarget.11826 (PMC5348339; doi:10.18632/oncotarget.11826)
Supplement: Supplementary file 1 [file oncotarget-07-80543-s001.pdf]

Targeting the receptor tyrosine kinase RET in combination with aromatase inhibitors in ER positive breast cancer xenografts

SUPPLEMENTARY FIGURES

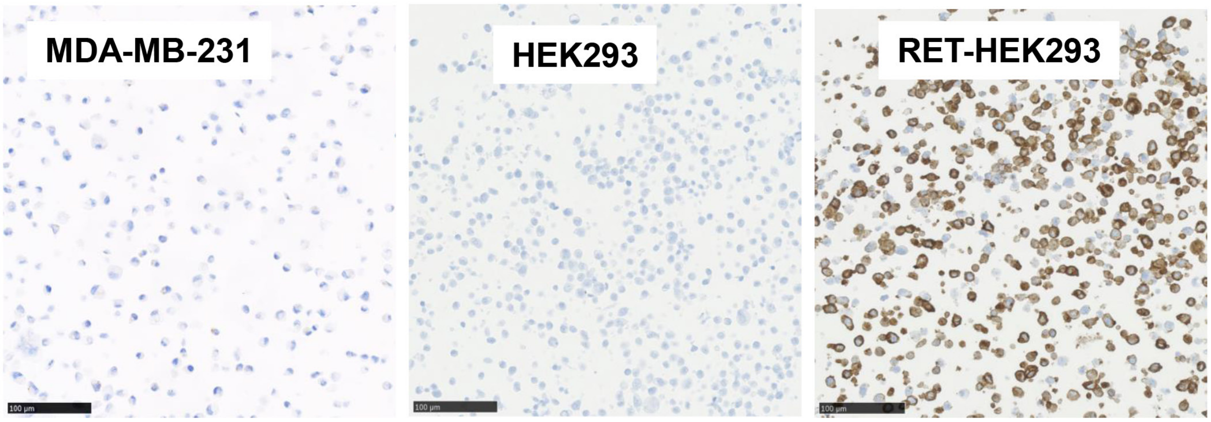

Supplementary Figure S1: Optimization of RET XP antibody for immunohistochemical staining of FFPE section of xenograft tumor. MDA-MB-231 and HEK293 (RET negative) and RET overexpressing HEK293 cells (RET positive) were cultured on tissue culture dishes, removed by scraping and pelleted. Cell pellets were fixed in 4% paraformaldehyde overnight and then subject to formalin fixation and paraffin embedding. 3 µm (micron) FFPE sections were cut and stained with the anti-RET XP antibody as described in Methods. Scale bar, 100 µm.

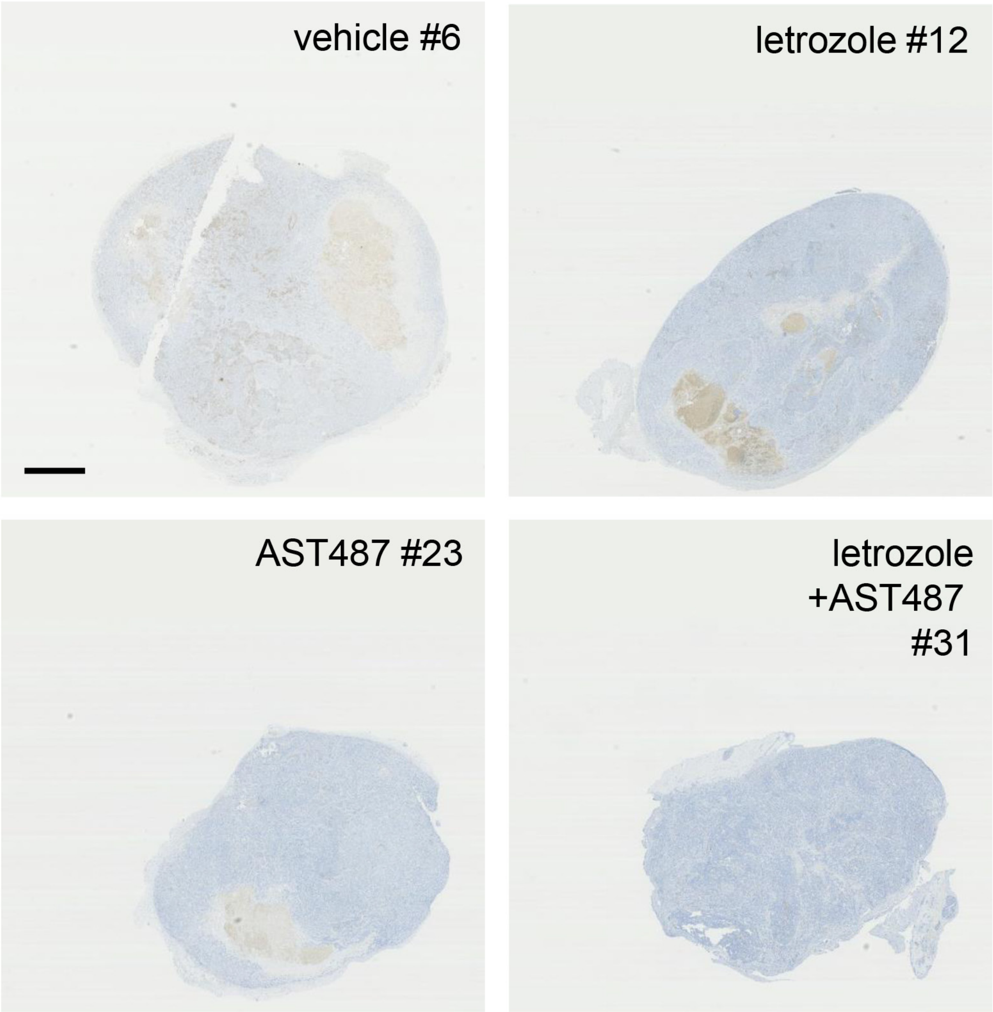

Supplementary Figure S2: Representative whole IHC RET slides of MCF7-AROM1 xenografts. Ovariectomized mice under androstenedione support were inoculated with MCF7-AROM1 cells. Following randomization, mice were treated daily with vehicle, letrozole, NVP-AST487 or letrozole plus NVP-AST487 as described in the Methods (n = 10 mice per group). Representative whole tumor IHC RET slides of vehicle, letrozole, NVP-AST487 and letrozole plus NVP-AST487-treated human MCF7-AROM1 xenografts. Scale bar, 1 mm.

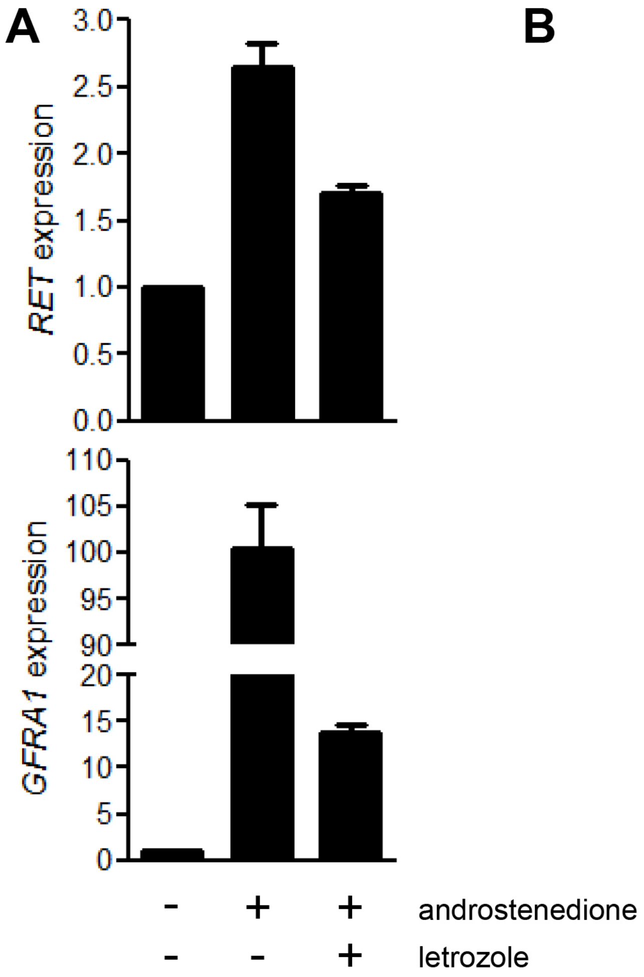

Supplementary Figure S3: BT474-AROM3 cells RET components and signaling characterization. A. Gene expression profiling of RET and GFRA1 in the BT474-AROM3 breast cancer cell line. **B.** Total cell protein extracts were subject to western blotting. NVP-AST487 blocks GDNF-induced RET downstream signaling. MCF7-2A cells were E2-deprived for 3 days, serum-starved overnight and treated with 100 nM NVP-AST487 for 90 min followed by GDNF stimulation (20 ng/ml) and GFRα1 (100 ng/ml) for 30 min. Total cell protein extracts were subject to western blotting.
